# Diabetes mellitus and HIV infection among active tuberculosis patients in Northwest Ethiopia: health facility-based cross-sectional study

**DOI:** 10.1186/s41182-021-00358-4

**Published:** 2021-08-28

**Authors:** Begna Tulu, Eden Amsalu, Yohannes Zenebe, Melkamu Abebe, Yeshimebet Fetene, Manamnot Agegn, Alemayehu Abate, Keerati Ponpetch, Teshome Bekana, Balako Gumi, Gobena Ameni

**Affiliations:** 1 Department of Medical Laboratory Sciences, Bahir Dar University, Bahir Dar, Ethiopia; 2 Aklilu Lemma Institute of Pathobiology, Addis Ababa University, PO Box 1176, Addis AbabaAddis Ababa, Ethiopia; 3 Department of Pediatric Nursing, Bahir Dar University, Bahir Dar, Ethiopia; 4 Sirindhorn College of Public Health Trang, Faculty of Public Health and Allied Health Sciences, Praboromarajchanok Institute, Ministry of Public Health, Nonthaburi, Thailand; 5 Department of Biomedical Sciences, Faculty of Public Health and Medical Science, Mettu University, Mettu, Ethiopia; 6 Department of Veterinary Medicine, College of Agriculture and Veterinary Medicine, United Arab Emirates University, PO Box 15551, Al Ain, United Arab Emirates

**Keywords:** Tuberculosis, Comorbidity, Diabetes mellitus, Risk factors, Northwest Ethiopia

## Abstract

**Background:**

The prevalence of diabetes mellitus (DM) is increasing globally and its comorbidity with tuberculosis (TB) is re-emerging, especially in low- and middle-income countries.

**Objective:**

The main aim of this study is to determine the prevalence of DM and HIV infection and their associated risk factors among active tuberculosis patients in Northwest Ethiopia.

**Methods:**

This hospital-based cross-sectional study was conducted between February 1st and June 30th, 2017 among active TB patients in two hospitals of Northwest Ethiopia. Two hundred and sixty-seven active TB cases aged 18 years or older were screened for diabetes using fasting blood glucose (FBG) test. Semi-structured questionnaires were used to collect demographic data, lifestyle habits and clinical data. Identification of pre-diabetes or diabetes in TB patients was achieved according to American Diabetes Association guidelines (2016).

**Results:**

Prevalence of DM and TB comorbidity was 11.5% (95% confidence interval, CI 7.8–15.2) compared to 24.9% (95% CI 20.1–30.1) for pre-diabetes. Prevalence of HIV/TB co-infection was 21.9% (95% CI 16.7–26.8). Risk of DM was higher in TB patients from a rural location (adjusted odds ratio, aOR 3.13, 95% CI 1.02–9.62, *p* = 0.046). Similarly, DM was higher in TB patients who have a family history of DM (aOR 4.54, 95% CI 1.31–15.68, *p* = 0.017). Furthermore, HIV/TB co-infection was identified as a predictor of DM comorbidity in active TB patients (aOR 5.11, 95% CI 2.01–12.98, *p* = 0.001).

**Conclusion:**

The magnitude of DM and pre-diabetes in active TB patients in Northwest Ethiopia was high, warranting collaborative efforts to improve screening and adopt better clinical management strategies for DM–TB comorbid patients. Furthermore, being rural residents, family history of DM and HIV/TB co-infection were found to associate with DM among TB patients, highlighting the importance of the above-mentioned risk factors in the clinical management of this comorbidity.

## Introduction

Diabetes mellitus (DM) is a chronic non-communicable disease which impacts the lives and wellbeing of our global population: the prevalence of DM in 2019 was estimated to be 9.3% (463 million people), which is predicted to rise to 10.2% (578 million) by 2030 and 10.9% (700 million) by 2045 [1]. In comparison, TB remains a major cause of ill health and is one of the top 10 causes of death worldwide. In 2018, an estimated 10 million people fell ill with TB, resulting in close to 1.2 million deaths among human immunodeficiency virus (HIV) negative individuals and 251,000 deaths among HIV-positive cases [2].

The high burden of TB is driven by risk factors like HIV, diabetes, substance misuse, advanced kidney disease, malnutrition and treatment with corticosteroids or immunosuppressants [3]. Diabetes patients have a three times greater risk of contracting TB compared to non-diabetic individuals [4]. Although the biological association of TB and DM is not fully understood, studies suggest that diabetes depresses immune responses, which in turn facilitates infection with *Mycobacterium tuberculosis* (*M. tuberculosis*) and/or progression to symptomatic disease. This is corroborated by the fact that diabetes is generally diagnosed before TB develops [5, 6]. Additionally, poor glycemic control adversely affects TB treatment outcomes with effects such as prolongation of culture conversion, treatment failure, relapse, and death [7].

According to studies conducted in different parts of the world, DM prevalence ranges from 0.1 to 44.5% in TB patients, as reviewed in [8]. In sub-Saharan Africa, including Ethiopia, the pooled prevalence of DM in TB patients was reported to be 9.0% [9] although it is estimated that 50% of the DM patients are not aware of their DM status in developing countries [10].

Epidemiological data on the magnitude of DM–TB comorbidity is important in order to control and prevent both diseases. In this regard, facility-based studies conducted in Ethiopia have indicated an increasing trend from 8.3% in South Eastern Amhara [11] to 13.5% in Eastern Ethiopia [12]. These studies also indicated factors like gender, older age, being pulmonary TB and having known family history with DM were associated with DM–TB comorbidity [11, 12]. Additional studies are required in different regions of Ethiopia in order to identify high-risk regions and promote proper patient management.

In order to curb the global epidemic of DM, TB and HIV, a collaborative framework for care and control of both diseases is important. More specifically, national programs, clinicians, and other care givers should be guided on how to establish effective and collaborative systems for both diseases at the organizational and clinical levels. In this regard, screening for DM and glycemic control in needed, as an essential part of TB patient management [10]. Data on the prevalence of DM and HIV in patients with active TB are important for proper health-care planning and resource allocation. Hence, the aim of this study was to estimate the prevalence of DM and HIV and its associated risk factors, among TB patients in Northwest Ethiopia.

## Materials and methods

### Study setting

**Fig. 1:**
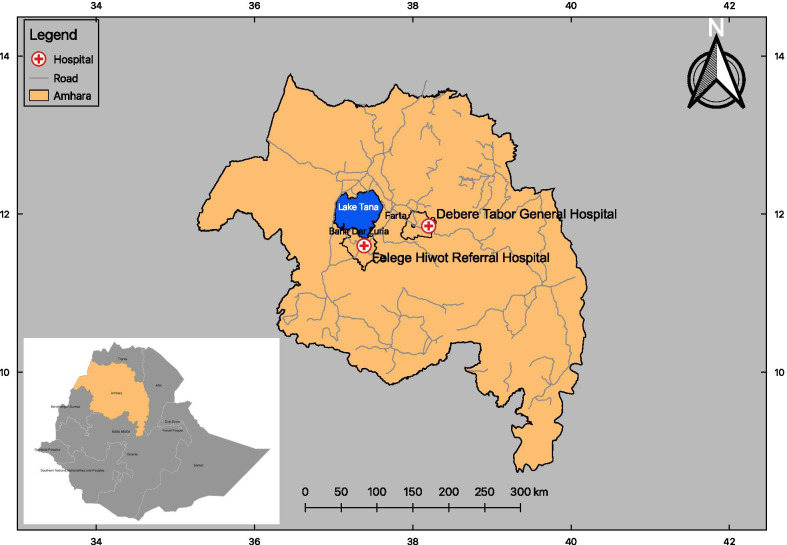
Map of the study area

The study was conducted in Felege Hiwot Referral Hospital (FHRH) and Debere Tabor General Hospital (DTGH) of Northwest Ethiopia, between February 1st and June 30th, 2017 (Fig. 1). FHRH is located in Bahir Dar, the capital of Amhara National Regional State, situated approximately 560 km from the capital city, Addis Ababa in the North of Ethiopia, which has a latitude and longitude of 11° 36′ N 37° 23′ E and an elevation of 1840 m above sea level. Based on the 2007 census conducted by the Central Statistical Agency of Ethiopia (CSA), Bahir Dar Special Zone has a total population of 221,991 (108,456 males; 113,535 females); 180,174 (81.16%) are urban inhabitants, the rest of population live in rural districts. Similarly, Debre Tabor Hospital is the main public Hospital in the South Gonder Zone, situated in Debre Tabor City which is located approximately 110 km from Bahir Dar, the capital of Amhara Region in the North East.

### Study design, population and sample size

This study was a hospital-based cross-sectional study conducted among active TB patients at FHRH and DTGH. All TB patients, new and those under anti-TB treatments attending both hospitals during the study period were included. Our sample size was determined using a single population formula and estimated by *p* = 8.3% [11] to obtain maximum sample size:

$$n = [Z\alpha /2(p)(1 - p)/d2],$$

where *Zα*/2 = level of significance, at 95% confidence interval = 1.96, *d* = marginal error 5%. Adding 20% non-response rate, the minimum sample size 140 individuals from both hospitals was considered.

In our two recruitment facilities, TB patients aged ≥ 18 years who were in treatment follow-up and were new active TB attendees, were considered for study inclusion during sample collection time. Diagnosis of TB was made based on national guidelines for the clinical and programmatic management of TB in Ethiopia [13]. Smear-positive pulmonary TB (PTB) was diagnosed in patients with at least two sputum smear examinations that were positive for acid-fast bacilli (AFB), or in patients that had one initial smear examination which was AFB-positive and additionally had radiographic abnormalities consistent with active TB, as determined by a clinician. Smear-negative PTB was diagnosed in patients with symptoms suggestive of TB, with at least three initial smear examinations that were negative for AFB by direct microscopy, and showed no response to a course of broad-spectrum antibiotics, or in patients that had three negative smear examinations by direct microscopy, radiological abnormalities consistent with PTB, and decision by a clinician to treat with a full course of anti-TB chemotherapy. The diagnosis of extra-PTB (EPTB) was made by identifying AFBs in organs other than lung, proven by at least one specimen with confirmed *M. tuberculosis*, or histological or strong clinical evidence consistent with active EPTB followed by a decision by a clinician to treat with a full course of anti-TB chemotherapy [16].

All patients diagnosed as having active TB were screened for DM through their history, previous medical records, and measurement of fasting plasma glucose (FPG) concentrations. The Mindray BS-200E chemistry analyzer was used for the determination of fasting plasma glucose level. Blood sugar status was classified based on the American Diabetes Association cut-off points. Accordingly, patients were classified into three groups: normal (70–99 m/dL), pre-diabetes (100–125 mg/dL), and diabetes (≥ 126 mg/dL) [19]. HIV sero-diagnosis was conducted according to the national test algorithm for Ethiopia following the manufacturer’s guidelines. For screening purposes, KHB (Shanghai Kehua Bio-engineering Co., Ltd) was used and positive samples were re-tested with STAT-PACK (Chembio HIV 1/2 STAT-PAK™ Assay, Chembio Diagnostic Systems, Inc., Medford, NY, USA). Discordant results were re-examined using the tiebreaker (Uni-Gold HIV, Trinity Biotech PLC, Co. Wicklow, Ireland). All TB patients were tested for HIV serology, which was part of the TB/HIV collaborative national program. Height and weight were measured using a digital weight scale and stadiometer. BMI was calculated, and weight status was classified using the WHO cut-off points.

### Data collections and quality assurance

An interview was conducted using structured questionnaire to collect information on socio-demographic characteristics and associated risk factors. Laboratory technologists/technicians in the TB laboratory and nurses in the TB treatment clinic were specifically trained and recruited to collect data.

Approximately 3 mL of venous blood was collected from patients by laboratory professionals. The sample was centrifuged and aspirated serum was stored at – 20 °C until later processing at Amhara Regional Health Research Laboratory Center. To assure good data quality, researchers were trained to collect data using a structured pre-testing questionnaire. Data entry and data checking was conducted using Epidata and was transferred to SPSS (version 21) for data analysis. The questionnaire was pre-tested on 30 randomly selected TB patients to ensure practicability, validity and interpretation of responses.

### Statistical analysis

Data entry, cleaning and analysis were performed using Statistical Package for Social Science (SPSS), version 21; New York, IBM Corp. Patients with non-conclusive blood glucose test results for DM diagnosis were excluded from the analysis. The main outcome variables were: proportions of patients with a diagnosis of TB-DM and TB-NDM. Categorical variables were expressed as proportions and Chi-square analysis was performed to compare proportions. Multivariate logistic regression analysis was performed to analyze the association of predictor variables with the outcome variable. A *p*-value < 0.05 was considered statistically significant. Effectiveness of the screening approach was estimated by the number of TB patients needed to screen (NNS) to get one additional new DM case, calculated by the reciprocal proportion of newly detected DM cases.

## Results

### Socio-demographic characteristics

Of the 280 TB patients who met inclusion criteria, 269 (96.1%) were included in the final analysis: six (2.1%) patients refused to participate in screening and 5 patients (2.0%) were excluded from the analysis because they did not have a confirmatory test result. Approximately 66.5% of participants were rural dwellers; the male-to-female ratio was 1.15, and the mean age of participants was 36.1 (SD 14.2; median age 35; range *X* to 62). The majority of the participants were married (59.5%) and 66.2% of participants had a family size of more than five (Table 1).

**Table 1:** Socio-demographic characteristics of the study participants

Characteristics	Total (%)	DM		HIV	
		Positive	Negative	Positive	Negative
Address					
Urban	90 (33.5)	5 (16.1)	85 (35.7)	31 (52.5)	59 (28.1)
Rural	179 (66.5)	26 (83.6)	153 (64.3)	28 (47.5)	151 (71.9)
Gender					
Male	144 (53.5)	19 (61.3)	125 (52.5)	29 (49.2)	115 (54.8)
Female	125 (46.5)	12 (38.7)	113 (47.5)	30 (50.8)	95 (45.2)
Age category					
18–30	126 (46.8)	9 (25.7)	117 (50.0)	28 (47.5)	98 (46.7)
31–40	61 (22.7)	13 (37.1)	48 (20.5)	13 (22.0)	48 (22.9)
> 40	82 (30.5)	13 (37.1)	69 (29.5)	18 (30.5)	64 (30.5)
Marital status					
Single	62 (23.0)	7 (22.6)	55 (23.1)	17 (28.8)	45 (21.4)
Married	160 (59.5)	19 (61.3)	141 (59.2)	31 (52.5)	129 (61.4)
Widowed or divorced	47 (17.5)	5 (16.1)	42 (17.6)	11 (18.6)	36 (17.1)
Family size					
< 5	178 (66.2)	15 (48.4)	163 (68.5)	42 (71.2)	136 (64.8)
≥ 5	91 (33.8)	16 (51.6)	75 (31.5)	17 (28.8)	74 (35.2)
Educational status					
No formal education	152 (56.5)	17 (54.8)	135 (56.7)	27 (45.8)	125 (59.5)
Primary (grade 1–8)	60 (22.3)	7 (22.6)	53 (22.3)	16 (27.1)	44 (21.0)
Secondary (grade 9–12)	26 (9.7)	3 (9.7)	23 (9.7)	5 (8.5)	21 (10.0)
College or University	31 (11.5)	4 (12.9)	27 (11.3)	11 (18.6)	20 (9.5)
Occupation					
Employee	31 (11.5)	4 (12.9)	27 (11.3)	11 (18.6)	20 (9.5)
Farmer	145 (53.9)	18 (58.1)	127 (53.4)	21 (35.6)	124 (59.0)
Merchant	23 (8.6)	2 (6.5)	21 (8.8)	9 (15.3)	14 (6.7)
Students	22 (8.2)	4 (12.9)	18 (7.6)	8 (13.6)	14 (6.7)
No job	48 (17.8)	3 (9.7)	45 (18.9)	10 (16.9)	38 (18.1)
Income per month in ETB					
< 1000.0ETB	113 (42.0)	15 (48.4)	98 (41.2)	23 (39.0)	90 (42.9)
1000.00–1999.00ETB	62 (23.0)	9 (29.0)	53 (22.3)	21 (35.6)	67 (31.9)
> 2000.00ETB	94 (34.9)	7 (22.6)	87 (36.6)	15 (25.4)	53 (25.2)

ETB: Ethiopian Birr, the exchange rate 1USD = 27.00ETB; DM: diabetes mellitus; HIV: human immunodeficiency virus

### Prevalence of DM and HIV among active TB patients

**Fig. 2:**
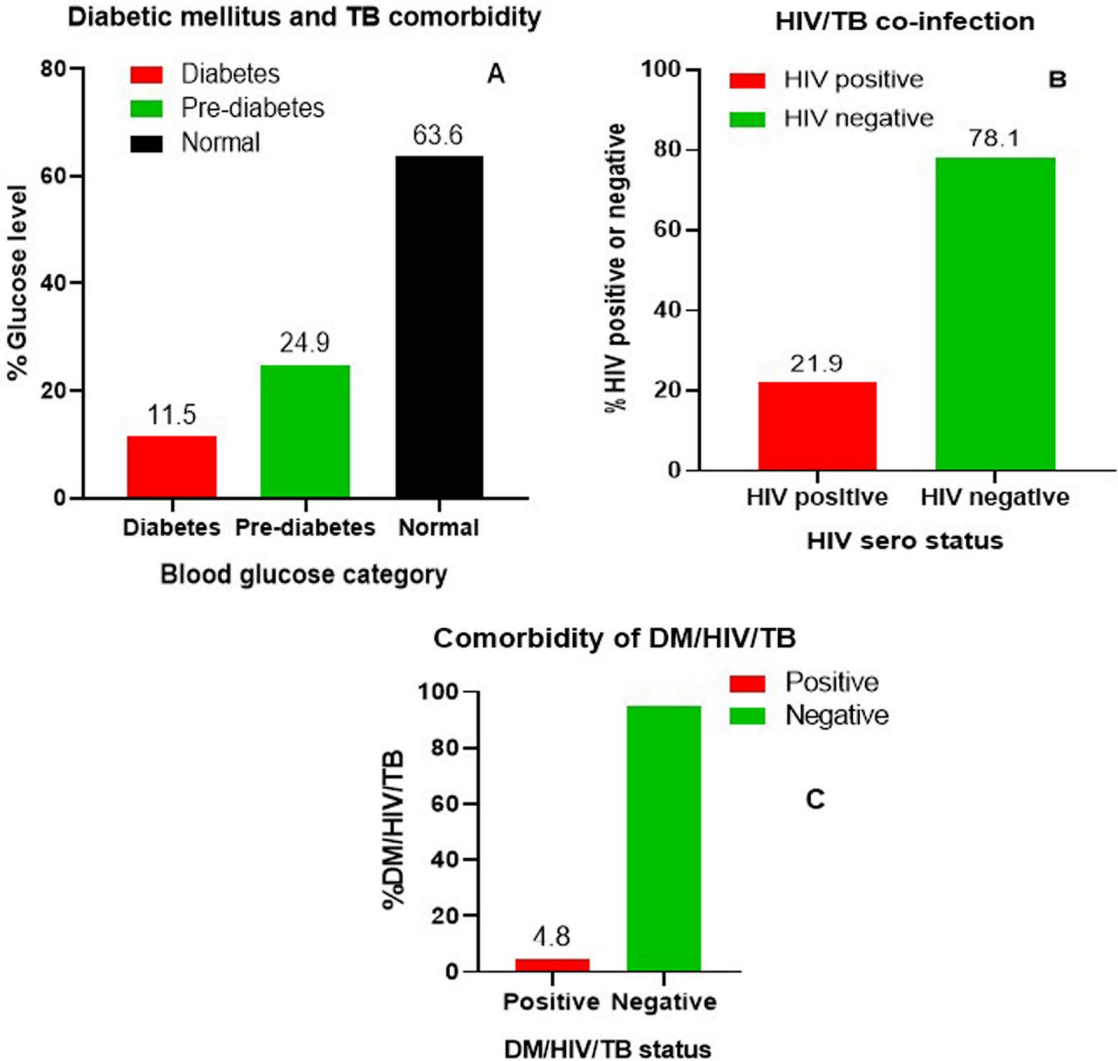
Prevalence of diabetes mellitus and HIV among TB patients. **A** Fasting plasma glucose level; **B** HIV–TB co-infection and **C** the triplets’ comorbidity (DM/HIV/TB) among TB patients. *DM* diabetes mellitus, *TB* tuberculosis, *HIV* human immunodeficiency virus

Based on the FPG analysis, prevalence of DM comorbidity (FPG ≥ 126 mg/dL) and impaired fasting glucose (FPG = 100–125 mg/dL) among active TB patients was 11.5% (31/269) and 24.9% (67/269), respectively. Prevalence of HIV/TB co-infection was 21.9% (59/269), and prevalence of DM/HIV/TB triple-infection was 4.8% (Fig. 2).

### Clinical characteristics and factors associated with DM, among TB patients

The majority (90.3%) of the DM–TB comorbid patients were new TB cases. Clinically, the majority of participants were EPTB (54.8%), followed by smear negative (41.9%). A total of 6.7% of TB patients reported that they had known family history of DM, of which 16.1% were found to be comorbid with DM. Furthermore, 64.5% of the DM–TB comorbid patients were found to be underweight (BMI < 18.5%) according to the WHO cut-off point (Table 2).

**Table 2:** Clinical characteristics and life style of TB patients

Characteristics	Total (%)	DMTB	NDMTB	HIV/TB	NHIV/TB
Type of TB patient					
New	243 (90.3)	28 (90.3)	215 (90.3)	53 (89.8)	190 (90.5)
Retreated	26 (9.7)	3 (9.7)	23 (9.7)	6 (10.2)	20 (9.5)
TB category					
Smear positive	33 (12.3)	1 (3.2)	32 (13.4)	13 (22.0)	20 (9.5)
EPTB	165 (61.3)	17 (54.8)	148 (62.2)	33 (55.9)	132 (62.9)
Smear negative	71 (26.4)	13 (41.9)	58 (24.4)	13 (22.0)	58 (27.6)
Known family history of DM					
Yes	18 (6.7)	5 (16.1)	13 (5.5)	3 (5.1)	15 (7.1)
No	251 (93.3)	26 (83.9)	225 (94.5)	56 (94.9)	195 (92.9)
BMI					
< 18.5	137 (50.9)	20 (64.5)	117 (49.2)	30 (50.8)	107 (51.0)
18.5–25.0	132 (49.1)	11 (35.5)	121 (50.8)	29 (49.2)	103 (49.0)
Any chronic diseases					
Yes	38 (14.1)	6 (19.4)	32 (13.4)	23 (39.0)	15 (7.1)
No	231 (85.9)	25 (80.6)	206 (86.6)	36 (61.0)	195 (92.9)
Active smoker					
Yes	6 (2.2)	1 (3.2)	5 (2.1)	0	6 (2.9)
No	263 (97.8)	30 (96.8)	233 (97.9)	59 (100.0)	204 (97.1)
Drink alcohol regularly					
Yes	107 (39.8)	13 (41.9)	94 (39.5)	21 (35.6)	86 (41.0)
No	162 (60.2)	18 (58.1)	144 (60.5)	38 (64.4)	

*BMI* body mass index, *EPTB* extra-pulmonary TB, *DM* diabetes mellitus, *TB* tuberculosis, *DMTB* DM among tuberculosis, *NDMTB* negative-DM among TB patients, *HIV/TB* human immunodeficiency virus infection among TB, *NHIV/TB* negative HIV infection among TB patients

In order to identify risk factors associated with DM comorbidity among active TB patients, a binary logistic regression analysis was conducted. The univariate analysis showed that rural residence; age greater than 34 years; HIV positivity, and known family history with DM were significantly associated with DM–TB comorbidity (*p* < 0.05).

In multivariable logistic regression analysis, residence, HIV co-infection, and known family history of DM were the independent predictors of DM–TB comorbidity (*p* < 0.05). TB patients who were rural residents were found to be 3 times more likely to be positive for DM (aOR 3.13, 95% CI 1.02–9.62, *p* = 0.046), compared to urban residents. HIV-positive TB patients were found to be 5 times more likely to be DM positive (aOR 5.14, 95% CI 2.00–13.25, *p* = 0.001), compared to HIV-negative patients. TB patients who had a known family history of DM were found to be more than 4 times more likely to be DM positive, compared to TB patients with no known family history of DM (aOR 4.54, 95% CI 1.31–15.68, *p* = 0.017) (Table 3).

**Table 3:** Factors associated with DM among TB patients in North Western Ethiopia

Characteristics	Total (%)	DMTB (%)	Crude OR (95% CI)	*p* value	aOR (95% CI)	*p* value
Address						
Rural	179 (66.5)	26 (83.9)	2.89 [1.07–7.79]	0.036	3.13 [1.02–9.62]	0.046
Urban	90 (33.5)	5 (16.1)	1		1	
Age in years						
18–34	134 (49.8)	10 (32.3)	1		–	–
> 34	135 (50.2)	21 (67.7)	2.28 [1.3–5.06]	0.042	–	–
TB category						
Smear positive	33 (12.3)	1 (3.2)	0.27 [0.04–2.12]	0.214	–	–
EPTB	165 (61.3)	17 (54.8)	1.95 [0.89–4.27]	0.094	–	–
Smear negative	71 (26.4)	13 (41.9)	1		–	–
Known family history of DM						
Yes	18 (6.7)	5 (16.1)	3.33 [1.09–10.08]	0.033	4.54 [1.31–15.68]	0.017
No	251 (93.3)	26 (83.9)	1		1	
HIV test result						
Positive	59 (21.9)	12 (38.7)	2.57 [1.16–5.65]	0.019	5.11 [2.01–12.98]	0.001
Negative	210 (78.1)	19 (61.3)	1		1	
BMI						
< 18.5	137 (50.9)	20 (64.5)	1.88 [0.86–4.09]	0.112	–	–
18.5–25	132 (49.1)	11 (35.5)	1		–	–

*aOR* adjusted odds ratio, *CI* confidence interval, *OR* odds ratio, *BMI* body mass index, *EPTB* extra-pulmonary TB, *DM* diabetes mellitus, *TB* tuberculosis, *DMTB* diabetic mellitus among tuberculosis

## Discussion

In this study, the prevalence of the DM and HIV infection and its associated risk factors were determined among active TB patients in Northwest Ethiopia. The prevalence of DM–TB comorbidity (11.5%) was higher than the national (8.5%) and the regional (9.0%) estimates [9], but lower than the global estimate (15.3%) [8]. Similarly conducted studies in Mali [14] and Serbia [15] have reported lower prevalences of DM–TB comorbidity (5.5% and 9.9%, respectively) than that of the present study, however there are reports of prevalences in excess of that we report: studies conducted in Ethiopia (13.5%) [12], Vietnam (13.7%) [16], South Korea (24.2%) [17], Mexico (25.2%) [18], Pakistan (39.6%) [19] and India (54.1%) [20]. The difference in socio-demographic characteristics of source populations studied, and the screening methods used could be responsible for such a wide range of DM prevalence among TB patients.

Pre-diabetes is characterized by an abnormally high blood glucose level, but one that is below the diabetic range. Pre-diabetic individuals have a higher risk of developing DM: each year approximately 5 to 10% of pre-diabetic individuals develop DM [21]. In the present study, pre-diabetes among TB patients was 24.9% lower than the prevalence reported in a previous study form Eastern Ethiopia (29.7%) [12]. The observed DM and pre-diabetes prevalence in the studied group deserves an integrated health service approach to address the burden of both DM and TB diseases.

There is a well-established association between advancing age and increasing risk of Type II DM [11, 12, 19]. Similarly, our univariate logistic regression analysis showed that those aged over 34 years had a significantly higher risk of DM–TB comorbidity. This could be explained by reports that advancing age is linked to immune impairment, which is a recognized risk factor for both DM and TB [22].

In this study, the majority of the participants were from rural areas and consequently, TB patients living in rural residences were found to be 3 times more likely to be positive for DM, compared to those living in urban residences. This finding is in contrast to a previous report [23], where participants in urban residences were found to be at higher risk of DM due to lifestyle differences. However, the higher risk of DM among participants living in rural residences in this study warrants further investigation using a longitudinal study design.

Having a family history of DM is a known risk factor associated with DM–TB comorbidity [11, 12, 16, 24]. Consistent with previous findings, the present study found the risk of DM to be 4 times higher among TB patients who had a family history of DM.

In this study, the prevalence of HIV among TB patients (21.9%) was higher than the prevalence of DM (11.5%) in TB patients. This finding is consistent with a previous study conducted in Ethiopia [11], but conflicts with a study conducted in India which reported the prevalence of HIV among TB patients to be 12.3% [25]. This may be due to the high burden of HIV in our study area. Similarly, HIV sero-positivity was a predictor of DM among TB patients in this study. The high prevalence of DM cases among TB/HIV cases may be linked to changes in quality of life and the adverse effects of anti-retroviral therapy (ART) drugs among HIV-positive patients. In order to enhance early detection and improve management of DM and HIV among TB patients, routine screening and a collaborative framework in existing health-care settings is recommended.

The main limitations of this study include: using random blood sugar and fasting blood sugar for DM diagnosis have lower sensitivity and specificity compared to an oral glucose tolerance test (OGTT). Furthermore, glycated hemoglobin (HbA1c) [26, 27] may underestimate the prevalence of DM. In addition, social desirability bias due to denial of reporting smoking, alcohol drinking and related habits may affect study findings related to behavioral risk factors for DM and TB comorbidity.

## Conclusion

In conclusion, this study reported a high prevalence of DM and HIV co-infection among active TB patients in Northwest Ethiopia. Thus, a collaborative approach should be implemented to improve the screening and management of DM among TB patients in TB clinics. Furthermore, rural residential status; a family history of DM, and HIV/TB co-infection were found to associate with DM–TB comorbidity, highlighting the importance of the above-mentioned risk factors in the clinical management of this comorbidity.

## Data Availability

All data generated or analyzed during this study are included in this published article.
